# A Case Study on the Microbiological Consequences of Short Supply Chains in High-Income Countries—The Consequences of Good Handling Practices (GHPs) in Vegetable Outlets in Portugal

**DOI:** 10.3390/foods14122036

**Published:** 2025-06-09

**Authors:** Ariana Macieira, Teresa R. S. Brandão, Paula Teixeira

**Affiliations:** CBQF-Centro de Biotecnologia e Química Fina–Laboratório Associado, Escola Superior de Biotecnologia, Universidade Católica Portuguesa, Rua de Diogo Botelho 1327, 4169-005 Porto, Portugal; tbrandao@ucp.pt (T.R.S.B.); pcteixeira@ucp.pt (P.T.)

**Keywords:** food safety, good handling practices (GHPs), vegetables, microbiological hazards, farmer’s outlets

## Abstract

Vegetables are commodities frequently sold in local markets and have been associated with foodborne outbreaks in short and local supply outlets worldwide. These outbreaks could potentially be mitigated through the implementation of good handling practices (GHPs) at points of sale. Numerous studies have assessed microbiological contamination in small-scale vegetable outlets in developing countries. In contrast, research on these risks in developed countries is comparatively scarce. However, with the increasing demand for vegetables, along with the increasing popularity of local markets, there is potential for an increase in foodborne outbreaks in developed countries. This study aimed to perform a microbiological assessment in local and short supply chain outlets of farmers in Portugal, as a case study, and to observe behaviors regarding GHPs in these outlets. The study was performed before and after the implementation of improved GHPs. This research employed quantitative analysis to measure the microbial load on vegetables, bench surfaces, and vendors’ hands. Additionally, a qualitative analysis was conducted to understand farmers’ behavior regarding GHPs using observational methods. Microbial hazards were detected in vegetables, on surfaces, and on hands both before and after the implementation of these practices, although the implementation of GHPs reduced the number of contaminations potentially associated with the practices used at the outlets. The results of this study highlight the importance of implementing GHPs in local and short supply chain markets for vegetables and fruits in developed countries, not only to protect consumers’ health, but also the farmers’ businesses.

## 1. Introduction

Fruit and vegetables are the most common commodities provided in short supply and local markets, due to their higher freshness and quality compared to those in longer supply chains [1,2]. Vegetables, especially those consumed raw, can pose potential threats to consumers if good handling practices (GHPs) are not followed by vendors. GHPs refer to a set of procedures and principles aimed at ensuring the safe and hygienic handling of food, particularly during post-harvest stages. These practices are designed to prevent contamination and spoilage, thus minimizing health risks and preserving food quality and safety. GHPs may include maintaining the personal hygiene of food handlers, cleaning and sanitizing equipment and containers, using safe water for rinsing, controlling temperature during storage and transport, and avoiding cross-contamination.

These commodities might be contaminated by various preharvest sources, such as manure and fecal contamination, contaminated compost and soil, inappropriate irrigation water, and agrochemicals, among others [3]. Improper handling practices during post-harvest activities can further contribute to microbial contamination of vegetables. Factors such as cross-contamination from rinse water, transport procedures, containers, contact surfaces, equipment, and food handlers can introduce pathogens into the produce [4,5]. Other factors that might increase post-harvest contamination levels are environmental conditions such as inadequate temperature, condensation drips, and lack of hygiene and sanitation practices [6,7]. As cross-contamination events are frequent, post-harvest operations management might be highly important to provide safe food to consumers [3,8]. Maintaining high standards of hygiene is important for consumers’ trust and the integrity of the farmer’s business [9]. High microbiological risks might be related to fruit and vegetables, according to Julien-Javaux et al. [10]. According to Sirsat et al. [11], several cases of bacterial contamination have been reported in farmers’ markets. Microbial contamination by *Escherichia coli* O157:H7, *Listeria monocytogenes*, *Salmonella* spp., *Bacillus cereus*, and *Clostridium perfringens*, among others, has been detected in fruit and vegetable samples [12,13]. Yeast and molds are known to degrade food [14] and, according to Instituto Nacional de Saúde Doutor Ricardo Jorge (INSA) [15], are considered to be indicator microorganisms of food hygiene, along with *Enterobacteriaceae* and aerobic mesophilic bacteria. Coagulase-positive staphylococci can also be an indicator of food contamination due to improper hygiene practices during handling [7]. According to Sirsat et al. [11], to prevent foodborne outbreaks, farmers and vendors need to implement GHPs. Numerous studies have investigated microbiological contamination and associated risks in small-scale and local vegetable outlets in developing countries. In contrast, such research is comparatively scarce in developed countries. This focus on developing countries is primarily due to insufficient infrastructure, inadequate handling practices, and limited food safety awareness [16]. However, the increasing consumption of vegetables, driven by health and environmental concerns, along with the growing popularity of local markets, could potentially lead to a rise in outbreaks in developed countries [13]. Therefore, the aim of this study was to evaluate the level of hygiene and food safety in small-scale and local vegetable outlets from farmers from the Northwest of Portugal. This case study serves as an example of assessing such risks in a developed country. The prevalence of hygiene indicators and pathogenic microorganisms was investigated during the selling period (beginning and end) in vegetables, bench surfaces where vegetables are handled, and the hands of vendors who handle the products, before and after the implementation of improved GHPs procedures and training sessions on food safety and GHPs. Simultaneously, handling behaviors were registered and related to the results of the microbiological assessment.

## 2. Material and Methods

### 2.1. Quantitative Approach

#### 2.1.1. Sampling

One kilogram of fresh vegetables was collected from four local outlets in the North of Portugal (Table 1), specifically in the Braga and Porto districts, at the beginning and end of the market. Along with this, hand and surface samples were also collected. From each composite vegetable sample (1 kg), as well as from surface and hand samples, three replicates were randomly prepared and analyzed independently. This procedure was repeated one year after farmers attended a GHP post-harvest training session, and after the implementation of GHP procedures in the outlet place. This study was developed in May 2022 and May 2023.

Outlets with more than one farmer used to mix the same type of vegetables from different farmers. There was no process of individual traceability at this point. The surfaces chosen to be analyzed were benches that the commodities could be in direct contact with. Surfaces were delimited (10 cm × 10 cm) and a random smear was performed. Vendors’ hands were also smeared on their palms and the back of both hands, fingers, and nails, and the swab was immersed in Ringer solution (10 mL) in between phases, and when hands were switched [15]. Samples were stored at 4 °C overnight before analysis.

#### 2.1.2. Microbiological Assessment

**Figure 1:**
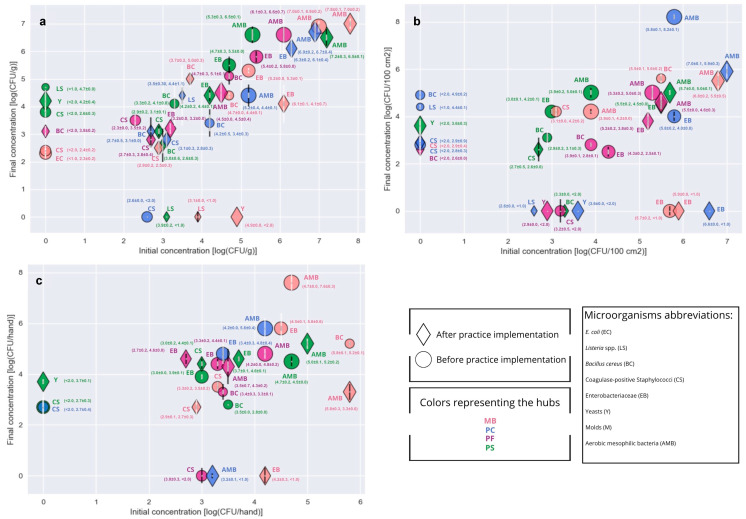
Microbial loading recorded in vegetables (**a**), surfaces (**b**), and vendors’ hands (**c**) before and after practice implementation, at the beginning and end of the selling. Black error bars represent the error of the samples collected at the beginning of the outlet (initial), and white error bars represent the error of the samples collected at the end of the outlet (final).

The studied microorganisms are described in Figure 1. Twenty-five grams of each unwashed produce were added to 225 mL of sterile buffered peptone water and homogenized in a Stomacher BagMixer (Interscience, Saint Nom la Brèteche, France) for 1 min, with a speed level 2. Serial decimal dilutions of the samples were prepared in Ringer’s solution for the microbial enumeration. Tryptone Bile X-glucuronide (TBX, Biokar diagnostics, Beauvais, France) agar was used to grow *E. coli* after incubation at 44 °C [17]. Agar Listeria, according to Ottaviani and Agosti (ALOA, bioMérieux, Marcy l’Etoile, France), was used for the enumeration of *Listeria* spp. after incubation at 37 °C for 48 h [18]. *B. cereus* was enumerated on Mannitol Egg Yolk Polymyxin Agar (MYP, Oxoid, Hampshire, UK), incubated at 30 °C for 18–24 h (or 48 h, if negative after 24 h), with typical colonies confirmed by the presence of hemolysis [19]. *C. perfringens* was enumerated on Tryptose Sulphite Cycloserine Agar (TSC, VWR Chemicals, Radnor, PA, USA) at 37 °C for 48 h under anaerobic conditions [20]. Coagulase-positive staphylococci were enumerated on Baird Parker agar (BPA, Biokar diagnostics) after incubation at 37 °C for 24 h [21]. Enterobacteriaceae were enumerated on RAPID Enterobacteriaceae Agar (Bio-Rad, Antibes, France) [22]. Yeasts and molds were enumerated on Rose Bengal Agar (RBA, Oxoid) and incubated at 25 °C for 5 days [23]. Aerobic mesophilic bacteria were counted on Plate Count Agar (PCA, Biokar diagnostics) after incubation at 30°C for 72 h [24]. The detection of *L. monocytogenes* and *Salmonella* spp. was also performed. For the detection of *L. monocytogenes*, 0.1 mL of incubated samples in half-Fraser bags were transferred to Fraser tubes (Merck, Darmstadt, Germany) and incubated at 37 °C for 24 h [25]. To detect *Salmonella* spp., after pre-enrichment in BPW for 24 h, 1 mL was transferred to Muller–Kauffmann Tetrathionate-Novobiocin broth (MKTTN, bioMérieux), and 0.1 mL to Rappaport–Vassiliadis soya peptone broth (RVS, bioMérieux), and incubated for 24 h at 37 °C and 41.5 °C, respectively [26].

Swab samples from the hands and surfaces were inoculated into 10 mL of sterile buffered peptone water, and then serial dilutions were performed. The microorganisms analyzed were the same as those analyzed in the vegetable samples.

Non-compliance criteria for vegetables and surfaces and hand contamination are present, respectively, in Table 2 and Table 3, and were based on the Guide to the interpretation of the results of microbiological tests on ready-to-eat foods and on surfaces in the food preparation and distribution environment, as published by INSA [15].

### 2.2. Qualitative Approach

**Figure 2:**
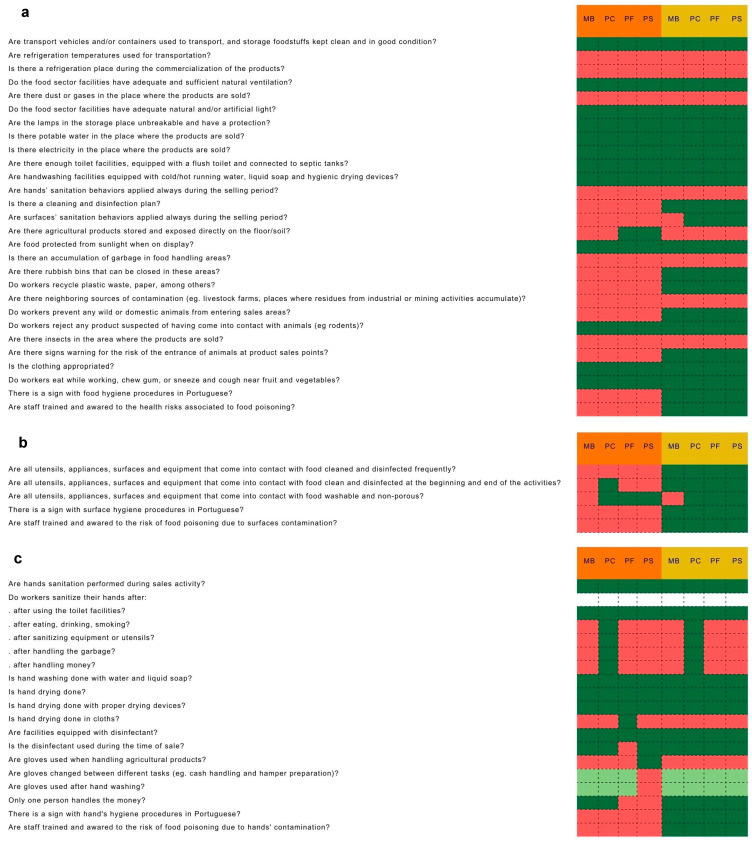
General practices (**a**), surface handling practices (**b**), and hand hygiene practices (**c**), adopted during vending, before (orange), and after (yellow) GHP implementation outlets, respectively (NO—Red block; YES—Green block; Not applicable—Light green).

**Figure 3:**
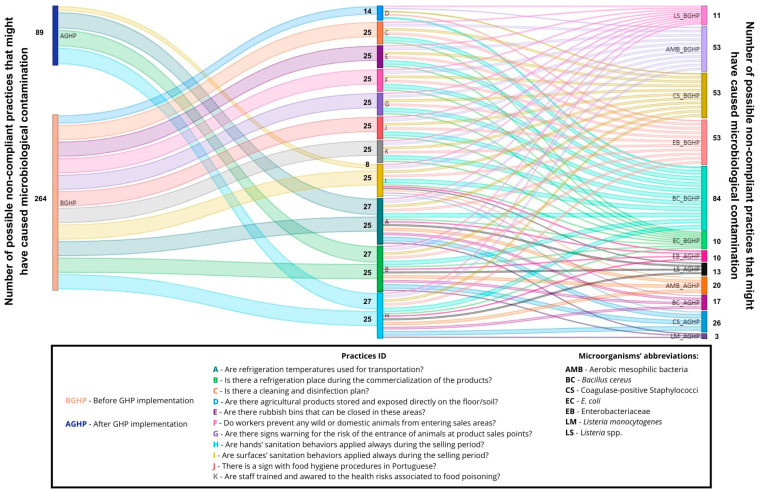
Sankey diagram representing the number of potential non-compliant practices in the outlets that may be implicated in bacterial contamination of vegetables, before and after practice implementation.

**Figure 4:**
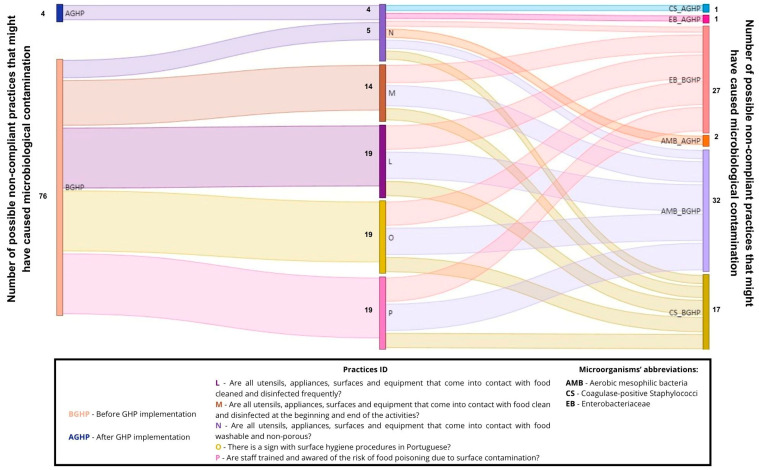
Sankey diagram representing the number of potential non-compliant practices in the outlets that may be implicated in bacterial contamination on surfaces, before and after practice implementation.

Qualitative research was performed according to Benke et al. [27]. Handling practices described in Figure 2, Figure 3 and Figure 4 were registered by the observer using a cellphone, by filling out a prewritten online form (Google Forms). Food, facility, equipment, and personal hygiene, including hand hygiene procedures, were previously written in lines in the form, being only necessary to fill only 3 possible columns (YES; NO; NOT APPLIED). There was an OBSERVATIONS line at the end of the form to write more information in case it was needed. Observation and data recording, when possible, were performed by keeping a distance from the selling spot, to avoid the observer influencing the farmer’s behavior. Before the development of this study, a declaration of consent was signed by the farmers who attended the study, as they agreed to be a part of the study. Farmers were aware that their behavior in the outlets would be followed and recorded, and that sampling would also be needed. A few days before the visit to the outlet, they were warned that the researcher would show up at the outlet to collect the data.

### 2.3. Data Analysis and Visualization

Data analysis and visualization were performed by a Python data visualization library named Seaborn v0.12.2, and Sankey diagrams were performed to establish a relationship between non-compliant GHPs and the total number of non-compliant microorganism levels on food, surfaces, and farmers’ hands at the outlets, by using Microsoft Power BI 2.119.986.0. A Sankey diagram is a type of flow diagram that visualizes the magnitude and direction of flows between categories—such as from specific GHP failures to different types of microbial non-compliances—using arrows whose width is proportional to the size of the flow.

## 3. Results

**Figure 5:**
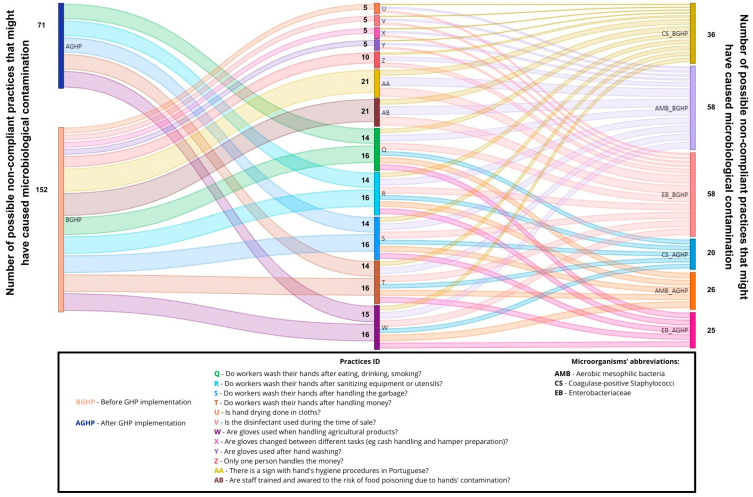
Sankey diagram representing the number of potential non-compliant practices in the outlets that may be implicated in bacterial contamination on vendors’ hands, before and after practice implementation.

The bacterial load found on surfaces, vegetables, and farmers’ hands before and after the implementation of the GHPs, and at the beginning and end of the outlets (2 to 3 h difference between samples), are shown in Figure 1. Observed practices and behaviors regarding GHPs in the outlets are shown in Figure 2. Table 4 shows the outlets that presented unsatisfactory results, according to INSA [15], at the beginning and end of the sales period, both before and after the implementation of GHPs. A relation was established between non-compliant GHPs and the total number (Initial + Final sampling) of non-compliant levels of microorganisms, collected on food, surfaces, and farmers’ hands in the four outlets (Table 2, Table 3 and Table 4; Figure 3, Figure 4 and Figure 5).

### 3.1. Vegetables

*Salmonella* spp. was not detected in any sample analyzed vegetables, but *L. monocytogenes* was detected in the initial sample of lettuce from the PS outlet, after practice implementation, although the loading was below 2.0 log (CFU/g).

Vegetable contamination by *Listeria* spp., aerobic mesophilic bacteria, coagulase-positive staphylococci, *Enterobacteriaceae*, *B. cereus*, and *E. coli* were detected before practice implementation (Figure 1a and Figure 3; Table 4). Before the implementation of GHPs, questionable concentrations of *Enterobacteriaceae* and aerobic mesophilic bacteria were detected in vegetable samples from MB and PF outlets at the beginning of the sales period. These issues persisted in the same outlets, with the addition of PS, at the end of the outlet, still prior to the implementation of GHPs. At the PF outlet, at the end of the market period, before GHPs were introduced, a potentially hazardous concentration of *B. cereus* was detected in the vegetables, posing a risk to consumer health. After the implementation of GHPs, none of the outlets presented questionable or potentially hazardous results for any microbiological parameter. However, some outlets continued to yield unsatisfactory results for *Listeria* spp., *B. cereus*, coagulase-positive staphylococci, *Enterobacteriaceae*, aerobic mesophilic bacteria, and *L. monocytogenes* (Figure 1a and Figure 3; Table 4).

The total number of non-compliant practices that might be related to microbial contamination, was higher before practice implementation (254) than after (89) (Figure 3).

### 3.2. Surfaces

*Salmonella* spp. was not found on any analyzed surface, but *L. monocytogenes* was detected on the surface of the PS vendor, at the beginning of the selling, after practice implementation. The cases of surface contamination were related to coagulase-positive staphylococci, *Enterobacteriaceae*, and aerobic mesophilic bacteria contamination before practice implementation (Figure 1b and Figure 4, Table 4). Surfaces were still non-compliant after practice implementation for aerobic mesophilic bacteria in all the outlets, and for samples from the final sales period of the PF and PS outlets, regarding *Enterobacteriaceae*; coagulase-positive staphylococci were detected at the beginning and end of the PS sales period (Table 4).

Only 4 cases of possible non-compliant practices might have contributed to microbial contamination after implementation practices, while 76 cases might have been responsible for contamination before implementation practices (Figure 4).

### 3.3. Vendors’ Hands

None of the hand samples were contaminated with *Salmonella* spp. nor *L. monocytogenes*. Lack of hand sanitation practices might contribute to the presence of hand contamination by coagulase-positive staphylococci, *Enterobacteriaceae,* and aerobic mesophilic bacteria, before and after practice implementation, although after GHP implementation, the number of outlets with non-compliant results for *Enterobacteriaceae* decreased (Figure 1 and Figure 5, Table 4). Furthermore, after practice implementation, the PC outlet stopped presenting non-compliant concentrations of coagula-positive staphylococci at the end of the sales period.

Before practice implementation 152 potential non-compliant causes could be related to microbial contamination, while after practice implementation, a reduction to 71 possible cases was recorded (Figure 5).

## 4. Discussion

This study combined a quantitative and a qualitative approach. The aim of implementing a quantitative approach was to assess the load of potential microbiological hazards on food, surfaces, and vendors’ hands at the beginning and end of the farmer outlets. A qualitative study was also performed, by using Benke observational techniques and recording the farmers’ behavior and practices regarding GHPs during the farmers’ selling period. According to Redmond and Griffith [28], Benke observational techniques might produce “more reliable data on food safety”, so these techniques were applied in this study, although the “Hawthorne Effect” might be a disadvantage of these sorts of techniques [29]. The “Hawthorne Effect” is a theory that proposes that when people are aware that are being a subject of observation, their behavior might change. The quantitative and qualitative approaches were performed before and after farmers attended a GHP post-harvest training session and the implementation of improved GHP procedures in farmer outlets. Some of the implemented hygienic procedures can be seen at https://rb.gy/xa73mn, accessed on 12 March 2025. All the information is in Portuguese, due to the context of this study. Ultimately, a relationship between both approaches was established by connecting the microbial load with farmers’ behaviors.

### 4.1. Vegetables

Although the *L. monocytogenes* concentration was below 2.0 log (CFU/g), in the initial sample of lettuce from the PS market, after practice implementation, this result was considered an unsatisfactory result, but not a potentially dangerous situation, according to INSA [15] (Table 2).

Levels of *E. coli* in vegetables were consistent with findings from studies by Bohaychuk et al. [30], Soendjojo [31], Sirsat and Neal [32], Wood et al. [33], Scheinberg et al. [34], and Kim et al. [35]. *Listeria* spp. *Listeria* spp. were also detected in vegetable samples from farmers’ markets by Scheinberg et al. [34] and Kim et al. [35]. Mgbakogu and Eledo [36] reported that contamination with *B. cereus* was also common in local vegetable markets. Similarly, Degaga et al. [37] analyzed vegetables from local markets in Ethiopia and found that 11% were contaminated with *Staphylococcus aureus*, mostly cabbage and lettuce.

The vendors’ outlets were attended mostly outdoors (Table 1), and according to Worsfold et al. [38] and Behnke et al. [27], farmers’ markets usually occurred outdoors, which might increase the probability of food contamination, due to environmental contamination, and the lack of essential facilities, such as the lack of temperature control tools during transportation and during the markets. In this kind of farmers’ market, cross-contamination was known as a higher safety risk due to poor personal, hand, utensil, and facilities sanitation [9,27,38], and due to many activities that farmers had to perform during the selling period, such as dealing with costumers, sanitizing and organizing the place, and dealing with other situations that might contribute to cross-contamination [39]. Some farmers used refrigeration chambers to cool and store their products after harvesting, but most transported their products immediately to the vending location after harvesting and washing. No refrigeration was used during transportation and vending due to a lack of funds and resources, despite all selling points having electricity access before and after the implementation of practices. According to Harrison et al. [40], 35% of farmers rarely or never refrigerated their produce during transport to the market. Although cooling was not performed in the outlets, vegetables were always kept away from direct sunlight and hot conditions. Before practice implementation, vendors let pets circulate near products [39] but after practice implementation, a prohibition sign was placed in the area of selling. Farmers maintained some non-compliant previous behaviors regarding GHPs, such as eating while working, chewing gum, or sneezing and coughing near vegetables.

Although vegetables may be subject to different environmental and climatic conditions from one year to the next, and seasonal variability can influence bacterial load, the primary objective of this study was not to compare contamination levels between types of produce or seasons. Rather, this study aimed to evaluate changes in handling practices at the same points of sale, as reflected in the microbial safety of the produce available at the time of sampling.

### 4.2. Surfaces

The lack of bench sanitation before starting selling the products, or a rare frequency of surface sanitation over the market, allied to the inexistence of hygienic procedures and training, might have contributed to surface microbiological contamination, before practice implementation [40]. Surface contamination after practice implementation could not be related to bacteria contamination, as those practices were improved by vendors, although there was only one practice that could be related, before and after practice implementation, to surface coagulase-positive staphylococci, *Enterobacteriaceae*, and aerobic mesophilic bacteria contamination, regarding the MB vendor, due to the fact that the bench where the vegetables were directly placed over the market was not easily washable and was porous (Figure 2b). After practice implementation, the MB bench was still being made of wood due to a lack of resources to replace the bench with a non-porous and washable one. McIntyre et al. [39] referred to the importance of vendors using appropriate washable and non-porous surfaces. According to Possas and Pérez-Rodríguez [41], contaminated surfaces might contaminate vegetables, increasing the risk of cross-contamination, although the viability of the cells might be threatened due to the lack of nutrients in surfaces. *L. monocytogenes* was detected on food and surfaces at the beginning of the PS vendor, after GHP implementation, and after surface sanitation at the beginning of the market, so *L. monocytogenes* cross-contamination from vegetables to surface might have occurred immediately after surface disinfection and vegetable manipulation, or perhaps the surfaces were not correctly cleaned, and the bacteria remained in the surface and the contamination was in the opposite way [42,43]. Microbial adhesion might be dependent on vegetables and surface roughness, cuticular wax, and other cuticular components [41,44,45].

### 4.3. Vendors’ Hands

Coagulase-positive staphylococci were detected before and after practice implementation, but these bacteria might be washed out by proper hand sanitation [27]. Before practice implementation, all the outlets were equipped with hand sanitizers, and vendors from three of the outlets used hand sanitizer during the selling, but after practice implementation, all of the vendors used hand sanitizers. The PS farmers used gloves during the selling period, but after practice implementation, this was finished. Although vendors sanitized their hands during the selling period by hand washing or by using disinfectant, after practice implementation, some non-compliant behaviors prevailed, such as vendors only washing their hands after going to the toilet, and farmers still not sanitizing their hands after eating, drinking, smoking, cleaning, or after handling money. McIntyre et al. [39] stated that regarding hand washing, the fact that there was hand washing equipment in the market, did not mean that hand washing might be performed. Allied to the existence of the equipment, might also be the compliance of the vendors’ behavior. Before practice implementation, more than one money handler was seen in the PS and PF selling points, which changed after practice implementation. The MB and PC vendors always had the same person handling the money, but the MB vendor consisted only of one vendor. Therefore, in this case, hand sanitation was crucial after money handling, and after practice implementation, the MB vendor always sanitized their hands after money handling. McIntyre et al. [39] reported that 90.9% of their vendors handle both food and money.

### 4.4. The Importance of the Implementation of GHPs and the Behavior of Microorganisms in the Outlets

Prior to practice implementation, several factors contributed to the observed behaviors and contamination: the limited number of vendors serving many clients, the lack of food safety practices, the absence of a food safety plan, and time constraints [38,40,46]. Following the implementation of practices, some non-compliant behaviors persisted, possibly due to the continuing shortage of farmers serving numerous clients simultaneously, and ongoing time constraints. Training and supervision are critical for implementation in these short food systems to mitigate risks and prevent foodborne illness [9,47]. In this study, most farmers had never received food safety training before implementing the practices, similar to the findings of Mohammad et al. [47]. As noted above, multiple factors could lead to cross-contamination at different points in the chain, contributing to higher levels of microbial contamination at the end of the sales period [48]. The implementation of GHPs and the training of vendors were critical in reducing hazards on surfaces, vegetables, and vendors’ hands, as demonstrated in Table 4, and by observing the relationship between GHPs and the microbial load in Figure 3, Figure 4 and Figure 5. However, significant hazards were found in the outlets even after the implementation of practices, possibly due to the persistence of previous behaviors.

According to Ko [9] and Arendt et al. [49], vendors might be aware of food safety procedures, although they might not always follow them. Possas and Pérez-Rodríguez [41] concluded that understanding the impact of certain food handling practices on cross-contamination throughout the supply chain was important to control and reduce the associated risks.

### 4.5. Limitations, and Future Perspectives

This study presents several limitations that should be acknowledged. Firstly, the sample size was limited to four local outlets in the North of Portugal, which may not fully represent the diversity of practices and microbiological risks present in other regions or in larger-scale markets. This study was conducted over two specific periods (before and after GHP implementation), which may not capture seasonal variations in contamination or handling practices. Additionally, the observational component may be subject to the “Hawthorne Effect”, where vendors alter their behavior because they are aware of being observed, potentially leading to an underestimation of non-compliant practices. The lack of individual traceability of produce from different farmers in outlets with mixed products could also confound the individual attribution of contamination sources. Finally, this study focused on a limited set of microbiological parameters and did not assess the presence of viruses or parasites, which can also be relevant in fresh produce safety.

Future research should consider expanding the sample size and including a broader geographic area to improve representativeness. Longitudinal studies covering different seasons could help assess the impact of environmental factors on microbiological risks. Incorporating molecular methods for source tracking and including a wider range of pathogens (including viruses and parasites) would provide a more comprehensive risk assessment. Further studies could also evaluate the long-term sustainability and effectiveness of GHP implementation, as well as consumer awareness and practices regarding food safety in local markets. Finally, exploring interventions tailored to specific challenges identified in diverse market settings would help optimize food safety strategies in short-supply chains.

## 5. Conclusions

Although this work was a case study carried out with only four farmers’ outlets, it highlighted and reinforced the importance of implementing GHPs in local and short-supply chain markets in developed countries. Before and after the implementation of practices, but especially before, there were instances where the microbial load on surfaces, vegetables, and vendors’ hands increased during the selling period, which could be a consequence of cross-contamination. Therefore, the implementation of practices was critical in eliminating or reducing the microbial load during sales at outlets. The implementation of GHPs should be a concern not only for consumers and local farmers, but also for local authorities and institutions. These bodies should help farmers understand, be aware of, and implement GHPs not only on the farm, but also at the point of sale, to increase consumer confidence and safety.

The findings from this Portuguese case study have implications beyond the country’s borders. They show that even in developed countries, where food safety standards are generally high, there is room for improvement in local and short supply chain markets. This research provides a model for other developed countries to assess and improve their own small-scale vegetable markets, and highlights the universal importance of implementing robust GHPs, regardless of a country’s overall level of development.

## Figures and Tables

**Table 1:** Number of members that attended the market, type of market, and type of commodity that was analyzed before GHP implementation and after GHP implementation.

Outlet ID	Outlet District	N° of Farmers in the Outlet	Outlet Places	Collected Vegetables (Before Practice Implementation)	Type of Vegetables (After Practice Implementation)
MB	Porto	1	Indoor	Lettuce (*Lactuca sativa*)	Lettuce (*Lactuca sativa*)
PC	Porto	2	Outdoor	Asparagus (*Asparagus officinalis*)	Asparagus (*Asparagus officinalis*)
PF	Braga	4	Indoor	Lettuce (*Lactuca sativa*)	Cucumber (*Cucumis sativus*)
PS	Braga	4	Indoor	Lettuce (*Lactuca sativa*)	Lettuce (*Lactuca sativa*)

**Table 2:** Reference values according to INSA [15] for vegetables.

Microbiological Parameters	Unsatisfactory	Unsatisfactory/Potentially Dangerous
*B. cereus* [log (CFU/g)]	3.0–≤5.0	>5.0
Coagulase-positive staphylococci [log (CFU/g)]	2.0–≤4.0	>4.0
*C. perfringens* [log (CFU/g)]	2.0–≤4.0	>4.0
Detection of *Salmonella* spp. (25 g)	NA	Detected
Detection of *L. monocytogenes* (25 g)	Detected	>2.0
	**Questionable**	**Unsatisfactory**
*E. coli* [log (CFU/g)]	1.0–≤2.0	>2.0
*Listeria* spp. [log (CFU/g)]	1.0–≤2.0	>2.0
*Enterobacteriaceae* [log (CFU/g)]	5.0–≤6.0	>6.0
Yeasts [log (CFU/g)]	5.0–≤6.0	>6.0
Molds [log (CFU/g)]	2.7–≤3.0	>3.0
Aerobic mesophilic bacteria [log (CFU/g)]	6.0–≤8.0	>8.0

**Table 3:** Maximum Admissible Values for microbiological parameters in surfaces [log (CFU/100 cm^2^)] and vendors’ hands [log (CFU/hand)], according to INSA [15].

		Maximum Admissible Values			
		*E. coli*	Coagulase-Positive Staphylococci	*Enterobacteriaceae*	Aerobic Mesophilic Bacteria
Surfaces in direct contact with the vegetables (benches)	After surface cleaning and disinfection (Initial)	<1.0	<2.0	<1.0	≤2.0
	Over the work period (Final)	<1.0	<2.0	<2.0	≤4.0
Vendors’ hands	After hand cleaning and disinfection (Initial)	<1.0	<2.0	<1.0	≤2.7
	Over the work period (Final)	<1.0	<2.0	<2.0	≤4.0

**Table 4:** Outlets’ IDs with non-compliant results, according to INSA [15], at the beginning and end of the outlets before and after practice implementation and respective microbiological parameters.

	Microbiological Parameters					
	*E. coli*	*Listeria* spp.	*B. cereus*	Coagulase-Positive Staphylococci	*Enterobacteriaceae*	Aerobic Mesophilic Bacteria
**Vegetables**						
Outlet ID with non-compliant results at the beginning of the outlet (before practice implementation)			MB, PC, PF, PS	PC, PF	MB ^(a)^, PF ^(a)^	MB ^(a)^, PF ^(a)^
Outlet ID with non-compliant results at the end of the outlet (before practice implementation)	MB	PS	MB, PC, PF ^(b)^, PS	MB, PF, PS	MB ^(a)^, PF ^(a)^, PS ^(a)^	MB ^(a)^, PF ^(a)^, PS ^(a)^
Outlet ID with non-compliant results at the beginning of the outlet (after practice implementation)		MB, PC, PS	MB, PS	MB, PC, PF, PS	MB, PC	MB, PC, PS
Outlet ID with non-compliant results at the end of the outlet (after practice implementation)		PC	MB, PC, PF	MB, PC, PF, PS	PC	MB, PC, PS
**Surfaces**						
Outlet ID with non-compliant results at the beginning of the outlet (before practice implementation)				MB, PF	MB, PC, PF, PS	MB, PC, PF, PS
Outlet ID with non-compliant results at the end of the outlet (before practice implementation)				MB, PC	PC, PF, PS	MB, PC, PF, PS
Outlet ID with non-compliant results at the beginning of the outlet (after practice implementation)				PS	MB, PC, PF, PS	MB, PC, PF, PS
Outlet ID with non-compliant results at the end of the outlet (after practice implementation)				MB, PC, PS	PF, PS	MB, PC, PF, PS
**Vendors’ hands**						
Outlet ID with non-compliant results at the beginning of the outlet (before practice implementation)				MB, PF	MB, PC, PF, PS	MB, PC, PF, PS
Outlet ID with non-compliant results at the end of the outlet (before practice implementation)				MB, PC, PS	MB, PC, PF, PS	MB, PC, PF, PS
Outlet ID with non-compliant results at the beginning of the outlet (after practice implementation)				MB, PS	MB, PF, PS	MB, PC, PF, PS
Outlet ID with non-compliant results at the end of the outlet (after practice implementation)				MB, PS	PF, PS	MB, PF, PS

^(a)^ Questionable results; ^(b)^ Potentially dangerous results.

## Data Availability

The original contributions presented in this study are included in the article; further inquiries can be directed to the corresponding author.
